# Phenotypic and Proteomic Analysis Identifies Hallmarks of Blood Circulating Extracellular Vesicles in NSCLC Responders to Immune Checkpoint Inhibitors

**DOI:** 10.3390/cancers13040585

**Published:** 2021-02-03

**Authors:** Davide Brocco, Paola Lanuti, Damiana Pieragostino, Maria Concetta Cufaro, Pasquale Simeone, Giuseppina Bologna, Pietro Di Marino, Michele De Tursi, Antonino Grassadonia, Luciana Irtelli, Laura De Lellis, Serena Veschi, Rosalba Florio, Luca Federici, Marco Marchisio, Sebastiano Miscia, Alessandro Cama, Nicola Tinari, Piero Del Boccio

**Affiliations:** 1 Department of Pharmacy, University “G. D’Annunzio” Chieti-Pescara, 66100 Chieti, Italy; maria.cufaro@unich.it (M.C.C.); laura.delellis@unich.it (L.D.L.); serena.veschi@unich.it (S.V.); rosalba.florio@unich.it (R.F.); piero.delboccio@unich.it (P.D.B.); 2 Department of Medicine and Aging Sciences, University “G. D’Annunzio” Chieti-Pescara, 66100 Chieti, Italy; paola.lanuti@unich.it (P.L.); pasquale.simeone@unich.it (P.S.); giuseppina.bologna@hotmail.it (G.B.); marco.marchisio@unich.it (M.M.); sebastiano.miscia@unich.it (S.M.); 3 Center for Advanced Studies and Technology (C.A.S.T.), University “G. D’Annunzio” Chieti-Pescara, 66100 Chieti, Italy; dpieragostino@unich.it (D.P.); lfederici@unich.it (L.F.); 4 Department of Innovative Technologies in Medicine and Dentistry, University “G. D’Annunzio” Chieti-Pescara, 66100 Chieti, Italy; detursi@unich.it (M.D.T.); grassa@unich.it (A.G.); 5 Clinical Oncology Unit, S.S. Annunziata Hospital, 66100 Chieti, Italy; pietro.dimarino@unich.it (P.D.M.); luciana.irtelli@tiscali.it (L.I.); 6 Department of Medical, Oral & Biotechnological Sciences, University “G. D’Annunzio” Chieti-Pescara, 66100 Chieti, Italy; ntinari@unich.it

**Keywords:** extracellular vesicles, biomarker, cancer immunotherapy, non-small cell lung cancer, immune checkpoint inhibitors

## Abstract

**Simple Summary:**

Purpose of this study was to investigate the prognostic and predictive role of blood circulating extracellular vesicles (EVs) in patients with advanced non-small cell lung cancer treated with immunotherapy. A newly optimized flow cytometry protocol was applied for identification and subtyping of blood circulating EVs in a total cohort of 59 NSCLC patients, which included 31 patients treated with anti-PD-1/PD-L1 agents and 28 patients treated with traditional chemotherapy. Our results show that pre-treatment concentration of blood circulating endothelial-derived EVs was correlated with overall survival and clinical response in patients treated with immunotherapy. Additionally, proteomic analysis of purified blood circulating EVs indicated differences in EV protein cargo between responders and non-responders to immunotherapy. These findings may pave the way to the identification of novel immunotherapy biomarkers in patients with advanced NSCLC.

**Abstract:**

Immune checkpoint inhibitors (ICIs) induce durable clinical responses only in a subset of advanced non-small cell lung cancer (NSCLC) patients. There is a need to identify mechanisms of ICI resistance and immunotherapy biomarkers to improve clinical benefit. In this study, we evaluated the prognostic and predictive value of circulating endothelial and leukocyte-derived extracellular vesicles (EV) in patients with advanced NSCLC treated with anti-PD-1/PD-L1 agents. In addition, the relationship between total blood circulating EV proteome and response to ICIs was investigated. An optimized flow cytometry method was employed for the identification and subtyping of blood circulating EVs in 59 patients with advanced NSCLC. Blood samples were collected from patients receiving anti-PD-1/PD-L1 inhibitors (*n* = 31) or chemotherapy (*n* = 28). An exploratory proteomic analysis of sorted blood EVs was conducted in a subset of patients. Our results show that a low blood concentration of circulating endothelial-derived EVs before treatment was strongly associated to longer overall survival (*p* = 0.0004) and higher disease control rate (*p* = 0.045) in patients treated with ICIs. Interestingly, shotgun proteomics revealed that EVs of responders to anti-PD-1 therapy had a specific protein cargo before treatment. In addition, EV protein cargo was specifically modulated during immunotherapy. We identified a previously unknown association between circulating endothelial-derived extracellular vesicle concentration and immunotherapy-related clinical outcomes. We also observed differences in circulating extracellular vesicle proteome according to anti-PD-1-based treatment response in NSCLC patients. Overall, these results may contribute to the identification of novel circulating biomarkers for rational immunotherapy approaches in patients affected by NSCLC.

## 1. Introduction

A growing understanding of cancer immune escape mechanisms led to the development and application of promising novel immunotherapy agents in NSCLC [1]. Despite advances in this field, a subgroup of patients bears tumors, which fail to respond or acquire resistance to the immunotherapeutic strategy [2]. Thus, there is a growing need for biomarkers that will enhance patient selection and understanding of immunotherapy response, helping to improve and personalize treatment regimens [3]. In this regard, obtaining tissue-related biomarkers is often challenging in advanced disease and expression of such biomarkers (e.g., cancer programmed cell death-ligand 1, PD-L1) display variability in time and space within the tumor, limiting their predictive value [4]. Circulating biomarkers may offer simpler, practical, and more representative tumor sampling in vivo [5]. Therefore, their identification is of paramount importance. 

Extracellular vesicles (EVs) are bilayer membrane-bound particles, released by almost all cells, which carry proteins, lipids and nucleic acids [6]. EVs are considered key mediators of intercellular communication and are involved in several physiological, or pathological processes, including cancer [7,8]. They play a pivotal role in remodeling the tumor immune microenvironment (TME) and in modulating the crosstalk among cancer cells, immune cells and other non-immune host cells taking part to the TME complexity [9]. In this regard, EVs released by cancer and tumor stromal cells can control anti-cancer immune system activity either by enhancing immune response against malignant cells or by promoting immunosuppressive TME modifications [10,11]. Thus, analysis of the molecular content of circulating EVs in cancer patients could inform about dynamics of tumor immune regulation and serve as tool for liquid biopsy in cancer immunotherapy [12,13,14]. With regard to NSCLC, two recent studies explored the value of EV-related RNA cargo in predicting response to ICIs [13,15]. Conversely, little is known about the predictive role of circulating EV subpopulations and protein cargo in NSCLC immunotherapy. 

In the present study, we applied a recently patented protocol using polychromatic flow cytometry (PFC), which allows EVs separation in fresh peripheral blood samples without pre-analytical EV enrichment procedures [16,17]. PFC was employed to characterize the immunophenotype of blood circulating EVs and to obtain a highly pure and intact EV pool for proteomic studies [17,18,19,20]. Regarding the immunophenotype, we investigated whether total circulating EVs, or those stemming from key TME-related cellular subsets, in particular endothelial cells and leukocytes, had a prognostic and/or predictive value in advanced NSCLC. The analysis of EV immunophenotype identified a previously unknown correlation between endothelial-derived extracellular vesicles and response to ICIs. Moreover, proteomic analysis revealed that ICI responders show distinct baseline and on-treatment EV protein cargo.

## 2. Materials and Methods

### 2.1. Patients

This prospective observational study enrolled adult patients with histologically or cytologically confirmed diagnosis of recurrent or de novo metastatic NSCLC and who were candidate to immunotherapy or chemotherapy for advanced disease. Patients were recruited from the Clinical Oncology Unit of the SS Annunziata Hospital in Chieti (Italy) from January 2016 to February 2020. A cohort of healthy controls was also enrolled in the study. All procedures performed in studies involving human participants were in accordance with the ethical standards of the 1964 Helsinki declaration and its later amendments or comparable ethical standard. The study was approved by the local ethics committee on 25 February 2016. All patients gave a written informed consent.

### 2.2. Blood Collection

Peripheral blood samples were collected at treatment baseline in the overall cohort. A second blood drawn was performed at 12 (+/−6) weeks after cycle 1 of therapy in NSCLC patients treated with ICIs. Blood samples were harvested in sodium citrate tubes (Ref 454387, Becton Dickinson Biosciences (BD), San Jose, CA, USA,) and processed within 8 h from collection.

### 2.3. Flow Cytometry Detection of Extracellular Vesicles

The EV staining was carried out as previously described [16,17,18,20] The reagent mix is detailed in Table S1. As we have previously published, the trigger threshold was set on the channel in which the lipophilic cationic dye (LCD), a general EV tracer, emits (allophycocyanin (APC) channel; threshold value: 200/262,144) [16,17,18]. The signal pulse height (H) was used both for scatter and fluorescent signals. EV scatter properties were established and validated by using both the Rosetta Calibration System (Exometry, Amsterdam, The Netherlands) and Megamix-Plus beads (Byocitex, Marseille, France), as described [16,17,18]. Fluorescence Minus One (FMO) and isotype controls were used to establish the gates on the basis of the respective nonspecific fluorescence [21]. Reagent-only, buffer-only and 1% Triton X-100 controls were also acquired. Compensation was assessed using CompBeads (BD) and single-stained fluorescent samples. Data were analyzed using FACSDiva v 6.1.3 (BD), FACSuite v 1.0.6.5230 (BD), and FlowJo v 10 (TreeStar, Ashland, OR, USA) software. Extracellular vesicle concentrations were obtained by volumetric count, using an instrument (BD FACSVerse™ flow cytometer) equipped by the volumetric count device. Such a function is largely used in flow cytometry to assess event counts [22,23].

### 2.4. Extracellular Vesicle Identification and Subtyping

**Figure 1:**
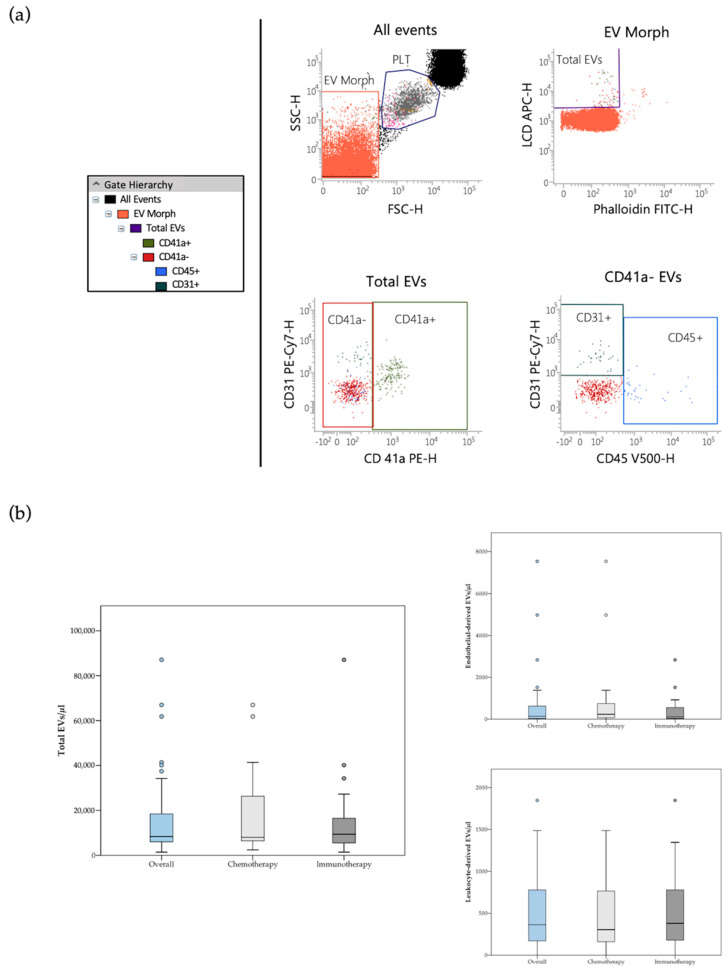
(**a**) Flow cytometry identification of total EVs, leukocyte-derived (CD45+ events) and endothelial-derived (CD41a−/CD31+/CD45− events) EVs in peripheral blood samples. The hierarchy of the gating strategy is represented. (**b**) Box plot diagram showing median blood concentration before treatment (horizontal black lines) of total EVs and EV subtypes in the overall population and in the two study groups. Extreme values are not shown.

The whole circulating EV population was identified as LCD+/phalloidin− events (active and intact EVs), falling in the scatter area containing events with physical parameters lower than that of platelets (Figure 1a). Detailed size and morphological features of blood circulating EVs detected by the LCD-based PFC protocol were described in previous reports [16,17]. According to Marchisio et al., the majority of the blood circulating LCD+/phalloidin-particles detected by this optimized PFC method presented a diameter larger than 160 nm (>90%) [16]. Phenotypic characterization of blood circulating EVs was based on selected transmembrane proteins, as suggested by the International Society for Extracellular Vesicles [24]. Leukocyte-derived EVs were identified on the basis of their positivity to CD45, while the EVs stemming from the endothelium were identified as CD41a−/CD31+/CD45− events (Figure 1a) [17,18]. As previously reported, direct comparison between the LCD-based PFC method with the Platelet-Free-Plasma (PFP) protocol promoted by the Society of Thrombosis and Haemostasis reported overlapping results in blood circulating CD41a+/CD31+ EV counting [16]. The PFC protocol here described avoids any pre-analytical enrichment steps, resulting highly convenient for translational purposes, therefore it was used in the present study.

### 2.5. Extracellular Vesicle Isolation by Fluorescence-Activated Cell Sorting

Extracellular vesicles were isolated as previously described [17,20,25]. A FACSAria III cell sorter (100 μm nozzle, BD Biosciences) was used to separate LCD+/Phalloidin-EVs from whole peripheral blood samples. The purity of EV isolated preparations was assessed by reanalyzing purified samples, as recommended, and purity was constantly > 90%.

### 2.6. Label-Free Proteomics of Circulating EVs

Pure EVs (2 × 10^6^) sorted from whole blood of six NSCLC patients were used for shotgun proteomics investigation. We normalized proteomic analysis with the number of EVs counted by fluorescent cell sorter for each group [17,18]. We pooled EVs samples as depicted in Figure S1. Filter-aided sample preparation (FASP) tryptic digestion protocol was performed and tryptic peptides were analyzed in triplicate by liquid chromatography tandem mass spectrometry (LC-MS/MS) using a Proxeon EASY-nLCII (Thermo Fisher Scientific, Milan, Italy) chromatographic system coupled to a Maxis HD UHR-TOF (Bruker Daltonics GmbH, Bremen, Germany) mass spectrometer, as we have previously described [17,18,20].

### 2.7. Proteomics Data Processing

Raw MS/MS data were processed using MaxQuant version 1.6.6.0 (Max-Planck Institute for Biochemistry, Martinsried, Germany). Peak lists were searched using Andromeda peptide search engine against the UniProt database (released 2018_04, taxonomy *Homo Sapiens*, 20,874 entries) Trypsin digestion was specified as digestion mode with a maximum of two missed cleavages. Carbamidomethylation of cysteines (C) was defined as fixed modification and used in protein quantification, while oxidation of methionines (M) and deamidation of asparagines and glutamines (NQ) were set as variable modifications as already reported [18]. Match-between-runs (MBR) algorithm was used to transfer the peptide identifications from one LC-MS/MS run to all others using its default settings (match window of 0.7 min and alignment time of 20 min). False discovery rate (FDR) at protein level was set at 3% in order to maximize characterization of EV-related proteins, while peptide level was set at 1% obtaining intensity-based absolute quantification (iBAQ) which was used for functional enrichment analysis.

### 2.8. Bioinformatics Analysis

Bioinformatics analyses were performed using Perseus software, version 1.6.10.50, (Max-Planck Institute for Biochemistry, Martinsried, Germany) uploading the identified protein groups generated by MaxQuant [26]. Data were log2 transformed in order to facilitate protein expression calculations. Site only, reverse, contaminant peptides and missing invalid values were removed from the dataset. The minimum number of valid values accepted was set at 2 in at least one clinical group. 

Gene Ontology and Pathway Analysis were performed using Ingenuity Pathway Analysis (IPA, Qiagen, Hilden, Germany) by loading the protein ratio as already reported [19]. Instead, the *p*-value is a measurement of the statistical overlap between the protein dataset and the genes or function categories, and the significance is attributed to *p*-value < 0.05. STRING (v. 11.0) analysis was used for evaluation of protein-protein interaction networks.

### 2.9. Statistical Analysis

Statistical analysis was performed using SPSS v23.0 (IBM SPSS, Chicago, IL, USA) and Medcalc v14.8.1 (MedCalc Software bvba, Ostend, Belgium). EV concentration were provided as median with 95% confidence interval. No assumption of normality of the data was formulated, therefore non-parametric tests were used for comparisons. Continuous data were compared using Mann–Whitney *U* test. The non-parametric Fisher’s exact test and Pearson’s chi-squared test were used to compare clinical variable distributions between EV groups. To find optimal cut offs, Cox proportional hazard regression was used to compute the predicted probabilities for total EV and each EV subtype concentration. Then, the receiving operator characteristic (ROC) curve with corresponding area under the curve (AUC) for each variable was calculated. Optimal cut-off values of ROC curves were estimated by Youden’s index. Median overall survival (OS) was calculated using the Kaplan–Meier (KM) curve estimator. Univariate and multivariate Cox proportional hazards models were applied to estimate hazard ratio (HR). The data cut off was set on August 2020. Clinical response in the chemotherapy and immunotherapy cohorts was evaluated according to RECIST v1.1 and iRECIST criteria, respectively. Disease control rate (DCR), defined as the percentage of patients who achieved complete response (CR), partial response (PR) and stable disease (SD), was analyzed and used to identify responder and non-responder groups. DCRs were compared using Fisher’s exact test. ROC curve of response (CR+PR+SD) vs. progression (PD) was generated to assess the predictive ability of baseline EV concentration. The relative EV count change was calculated as % change ({[EV concentration week 12/EV concentration week 0] − 1} ∗ 100) and divided in two groups: decreasing (≥25% decrease) and stable or increasing (<25% decrease to <25% increase or ≥25% increase) EV concentration. The SPSS biased-corrected and accelerated bootstrap method with 1000 bootstrap samples and 95% confidence interval was used for internal validation. A two tailed *p*-value of <0.05 was considered statistically significant.

## 3. Results

### 3.1. Patients Characteristics

Baseline demographic and clinical characteristics of all enrolled NSCLC patients (*n* = 59) are summarized in Table S2. A total of 31 patients were treated with PD-1 or PD-L1 inhibitor monotherapy (pembrolizumab [*n* = 22]; atezolizumab [*n* = 5]; nivolumab [*n* = 4]) while 28 patients were treated with chemotherapy [platinum-based doublet (*n* = 25); docetaxel [*n* = 2]; carboplatin [*n* = 1]). Median follow-up time was 8.0 (95% CI 5.0–11.0) months; 15 patients (25.4%) were alive at the time of the analysis. One-year OS was 39% for the overall population, 48% for patients treated with ICIs and 30% for the chemotherapy group. Tumor response was evaluable in 57 of 59 patients. The percentage of patients who achieved complete response, partial response and stable disease was 49.1% in the overall cohort, 53.6% in the immunotherapy group and 46.4% in the chemotherapy group.

### 3.2. EVs Frequencies

Median pre-treatment blood concentrations of total, leukocyte-derived, as well as endothelial-derived EVs are reported in Table S3. Total and endothelial-derived EV concentrations were compared between the overall population of patients and a cohort of age and sex-matched healthy controls (*n* = 27) (Table S4). Of note, median total and endothelial-derived EV concentrations were significantly lower in the control group [median total EVs/μL (95% CI) = 4045 (2503–6243); median endothelial-derived EVs/μL (95% CI) = 62 (42–107) as compared to the cancer cohort [median total EVs/μL (95% CI) = 8414 (6647–14350); median endothelial-derived EVs/μL (95% CI) = 146 (73–385)]. No significant difference in total and single subtype EV concentration at baseline was observed between patients treated with chemotherapy and patients treated with PD-1/PD-L1 inhibitors (Figure 1b and Table S3). Notably, in the immunotherapy cohort, analysis of the follow-up samples revealed a significant decrease of endothelial- and leukocyte-derived EV concentration following immunotherapy (Table S5). In particular, a greater than 60% decrease in endothelial- and leukocyte-derived EV levels was detected during treatment. 

### 3.3. Circulating Endothelial-EV Concentration Is Associated with Overall Survival

We investigated whether pre-treatment EV levels were correlated with survival following treatment. Cut-off values for survival analysis were calculated for total EVs and single EV subpopulations from the whole patient cohort (Total EVs cut-off = 14,360 EVs/μL; Leukocyte-EVs cut-off = 169 EVs/μL; Endothelial-EVs cut-off = 94 EVs/μL), as described in Methods. The same cut-off points calculated from the overall patient population were then used in the two separated treatment cohorts. We performed univariate and multivariate Cox proportional hazards regression analysis to evaluate the association between baseline EV concentration and survival. Different variables including age, number of metastatic sites, ECOG PS, tissue PD-L1 expression and line of therapy were evaluated as potential risk factors of OS using univariate Cox proportional hazards regression analysis and significant predictors of OS were included in the multivariate analysis.

Estimated HRs for whole circulating EVs and single EV subtypes group are reported in Table 1 for the immunotherapy cohort and Table S6 for the overall population and chemotherapy cohort. Both in the overall population and in the immunotherapy cohort univariate Cox proportional hazards regression analysis showed a significant reduction in the risk of death in patients with endothelial-derived EV concentration below the relative cut-off of 94 EVs/μL, as compared to patients with higher blood endothelial-derived EV concentration (Table 1 and Supplementary Table S6). These results were confirmed by multivariate Cox regression analysis. Conversely, univariate and multivariate Cox proportional hazards regression survival analysis did not reveal statistically significant association between OS and endothelial-derived EV blood concentrations before treatment in patients treated with a chemotherapy regimen (Supplementary Table S6). Risk of death did not significantly differ between patients with higher and lower levels of total blood EVs, as well as leukocyte-derived EVs in the study cohorts (Table 1 and Supplementary Table S6). All the results obtained by univariate and multivariate analyses were confirmed via bootstrap validation. 

**Figure 2:**
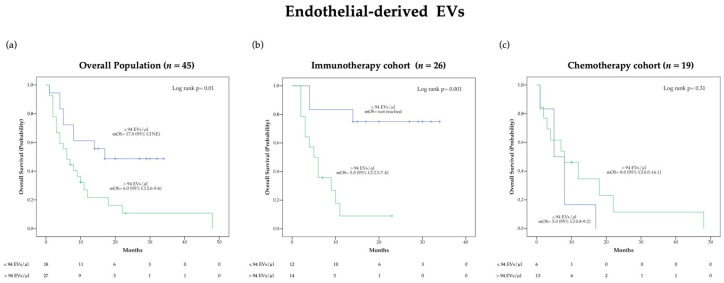
Kaplan-Meier (KM) curves examining the relationship between overall survival and blood circulating endothelial-derived EV concentration before treatment in the overall population (**a**), the immunotherapy cohort (**b**) and the chemotherapy cohort (**c**).

OS was also evaluated using Kaplan-Meier (KM) survival curves (Figure 2 and Supplementary Figure S2). Of note, a significant difference in median OS (mOS) according to pre-treatment endothelial-derived EV concentration was observed in the overall population (*p* = 0.01) as well as in the immunotherapy cohort (*p* = 0.001) but not in the chemotherapy cohort (Figure 2). Specifically, patients presenting endothelial-derived EV concentrations lower than 94 EVs/μL before immunotherapy were characterized by an extremely favorable outcome with mOS not reached at the time of analysis cut-off, as compared to a mOS of 5.0 (95% CI 2.5–7.4) months in the group with higher blood EV level (Figure 2b). Distribution of clinical characteristics according to baseline endothelial derived-EV concentration in patients treated with ICIs is reported in the Supplementary Table S7.

### 3.4. Circulating Endothelial-EV Concentration Is Associated with Disease Control Rate

**Figure 3:**
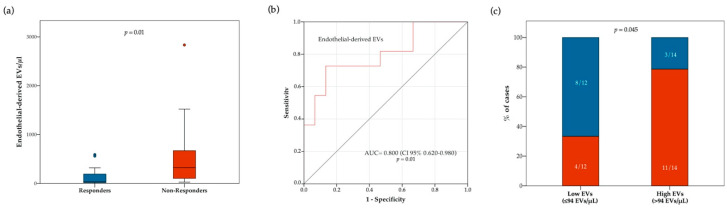
Relationship between response to ICIs and blood circulating endothelial-derived EV concentration at treatment baseline. In panel (**a**), boxplot diagram showing difference in median endothelial-derived EV concentration (horizontal black lines) between responders and non-responders. Two-tailed Mann–Whitney test was performed, and *p* value is shown. In panel (**b**), receiver operating characteristic (ROC) curve illustrating predictive abilities on ICI response of blood circulating endothelial-derived EVs. In panel (**c**), histograms showing difference in disease control rate (DCR) according to baseline endothelial-derived EV concentration (cut-off point: 94 EVs/ μL). Blue and red bars indicate proportion of responders and non-responders, respectively. Fisher’s exact test was used to compare the two groups.

We then explored the relationships between EV concentrations and treatment response. Blood concentration of total and subtype EVs at baseline were compared between responders and non-responders (Table S8). Notably, the overall population of responders had lower median pre-treatment concentrations of total EVs (*p* = 0.03) and endothelial-derived EVs (*p* = 0.02) (Table S8). However, approximately 3-fold lower median pre-treatment concentrations of total EVs (*p* = 0.03) were observed only in responders of the chemotherapy cohort, while no difference was observed in the immunotherapy cohort (Table S8). Conversely, approximately 10-fold lower median pre-treatment concentrations of endothelial-derived EVs were observed only in responders of the immunotherapy cohort (*p* = 0.01), but not in the chemotherapy cohort (Figure 3 and Table S8). As shown in Figure 3b, receiving operator characteristic (ROC) curve analysis confirmed a correlation between blood circulating endothelial-derived EVs and immunotherapy response (AUC = 0.800 [CI 95% 0.650–0.980]; *p* = 0.01). We also compared the proportion of responders and non-responders to anti PD-1/PD-L1 agents in patients with higher or lower endothelial-derived EVs at baseline (cut-off: 94 EVs/μL). The proportion of responders was higher in patients with low, as compared to those with high endothelial-derived EVs (66.7% vs. 21.4% respectively; *p* = 0.045) (Figure 3c). We further analyzed the relationship between response to ICIs and variations in EV concentration between baseline and on-treatment samples. In this regard, no significant differences in DCR were observed between patients with stable or increasing and decreasing total, as well as single subtype EV concentrations (Supplementary Figure S3).

### 3.5. Proteomic Analysis Reveals Specific Protein Cargo in Responders vs. Non Responders

Two million EVs were isolated by fluorescence activated cell sorter and digested for proteomic characterization to explore the proteomic profile of EVs in 3 responders and 3 non responders (Supplementary Figure S1). The following samples were analyzed: baseline EVs from responsive pre-treatment patients (RB), baseline EVs from unresponsive pre-treatment patients (NRB), post-treatment EVs from patients responding to PD-1 inhibitor (RP) and post-treatment EVs patients who did not respond to PD-1 inhibitor (NRP). The list of quantified proteins present in at least two out of three analytical replicates for each sample group is reported in Supplementary Table S9, sheet “Quantification”. Protein numbers were consistent with those obtained in previous analyses of EVs purified by PFC [20].

**Figure 4:**
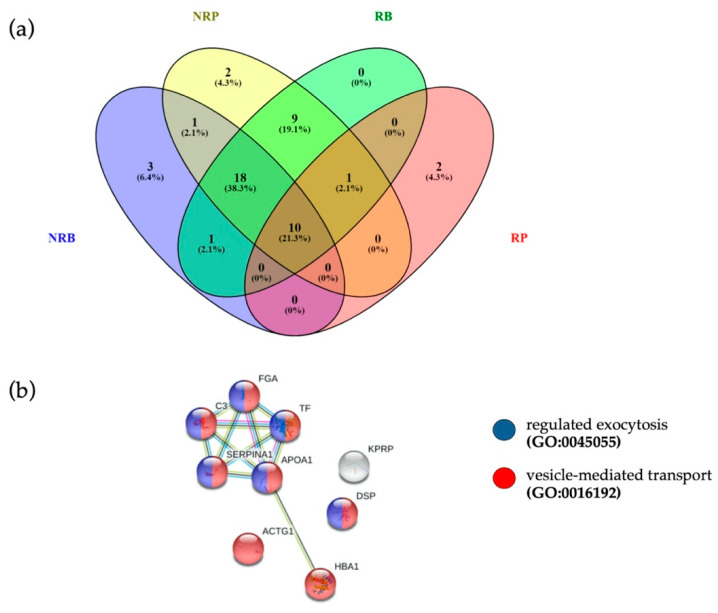
(**a**) Venn diagram of quantified proteins in RB, NRB, RP and NRP. (**b**) The 10 proteins common to four different clinical conditions were associated to “vesicle-mediated transport” (FDR = 2.27 × 10^−5^, red dots, PPI enrichment *p*-value = 9.53 × 10^−7^) and “regulated exocytosis” (FDR = 6.35 × 10^−5^, blue dots), terms coherent with EVs.

In search for potential pre-treatment biomarkers of response, we verified whether EVs of responders at baseline were loaded with specific proteins, by comparing EV protein cargo of RB and NRB. Ten proteins were detected only in pre-treatment EVs of responding patients, as reported in Supplementary Figure S4 and Supplementary Table S10. Overall, in the four groups analyzed we quantified at baseline 33 proteins in EVs from not responders (NRB) and 39 proteins in EVs from responding patients (RB), while post-treatment 41 EV proteins in not responders (NRP) were quantified and only 13 EV proteins in responders (RP) (Figure 4a).

### 3.6. Anti-PD1 Treatment Modulates a Subset of EV Proteins Involved in Immune Function

Intriguingly, a subset of EV proteins were differently modulated during treatment between responders and non-responders. (Figure S5). Relative on-treatment modification of protein expression was calculated as fold-change from baseline in LOG-scale, as depicted in Figure S5. STRING analysis (PPI enrichment *p*-value = 5.61 × 10^−7^) revealed that these 10 EV-proteins were related to “neutrophil degranulation” (FDR = 5.58 × 10^−9^; green dots), “defense response” (FDR = 5.58 × 10^−9^; blue dots) and “immune response” (FDR = 2.1 × 10^−9^; red dots) (Figure S5 and Table S12).

### 3.7. Anti-PD-1 Treatment Modulates Pathways Involved in Immune Function

The intensity-based absolute quantification (iBAQ) value of individual proteins detected was used as a quantification parameter, and the ratio used for functional analysis is reported in Supplementary Table S9, sheet “Fold Change”. Ratios of quantified proteins were subjected to functional reclassification analyses through IPA bioinformatics tool to study both the responders (RP vs. RB ratio) and non-responders (NRP vs. NRB ratio). The two functional analyses were compared through IPA, as reported in the workflow in Supplementary Figure S1 highlighting the differences between the two clinical scenarios. 

**Figure 5:**
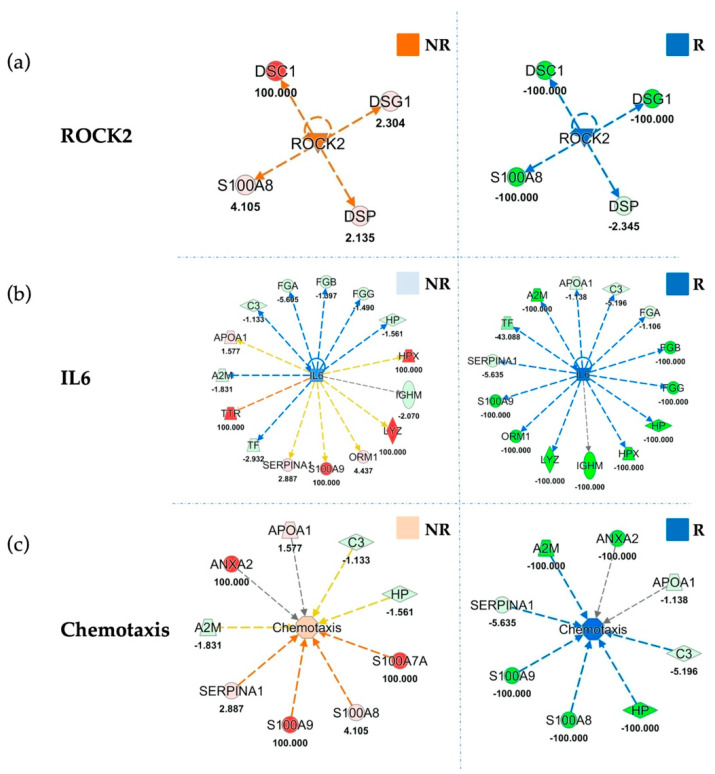
Functional Comparison Analysis between activated and inhibited pathways in sorted EVs from not responding (NR) and responding (R) patients, as compared to their respective baselines. Panel (**a**) highlights *ROCK2* in NR and R sorted EVs compared to their baselines. Panel (**b**) shows *IL6* modulation in NR and R EVs. Panel (**c**) reports the same comparison for “Chemotaxis” function. Overall ROCK2, IL6 and Chemotaxis are inhibited (blue) in responders, as compared to baseline, whereas in non-responders they result activated (orange) or unchanged (light blue). Red and green shapes represent increased or decreased measurements of identified proteins, respectively, whose fold change value is reported in the figure. Color key and symbols are reported in Supplementary Figure S8.

Upstream analysis indicated inhibition of the immune system regulator Interleukin-6 (IL6) (Figure 5b) in responders, with an inhibition z-score of −3.52. On the other hand, IL6 was almost unchanged in the EVs of patients who did not derive clinical benefit from treatment (z-score = −0.93). Accordingly, our data showed strong inhibition of Signal transducer and activator of transcription 3 (STAT3) in RP (z-score = −2.94) that is in accordance with the described IL6 inhibition, which is its regulator, as reported in Figure S6b. Moreover, our upstream analysis showed inhibition of another upstream regulator related with immune function PR domain zinc finger protein 1 (PRDM1) (z-score = −2,53), as reported in Table S11, in RP with respect to baseline, while PRDM1 was unmodulated in NRP (z-score = 0.053). Similarly, upstream analysis indicated a strong inhibition of another protein involved in immune escape, oncostatin M (OSM) (z-score of −2.45), in EVs sorted from RP patients compared to their baseline. Conversely, OSM was not modulated at all in unresponsive patients (z-score = 0.07) (Table S11). In addition, upstream analyses show an inhibition (z-score = −1.982) of the EV release modulator Rho-associated protein kinase-2 (ROCK2) in responding patients, and its activation (z-score = 1.982) in unresponsive patients following treatment (Figure 5A).

Furthermore, among most significant results of the downstream analyses, the inhibition of “Chemotaxis” (z-score = −2.37) and “Chemotaxis of myeloid cells” (z-score = −2.12) in EVs of responding patients (Figure 5c; Table S11). The inhibition of chemotaxis was corroborated by the effect of treatment in modulating metals efflux in cells (Figure S7).

## 4. Discussion

Treatment with immune checkpoint inhibitors led to significant improvement of clinical outcomes in NSCLC patients [27]. However, a substantial percentage of patients do not receive any clinical benefit and may develop severe adverse events. Thus, discovery of biomarkers that will predict response to cancer immunotherapy agents is crucial to provide improved and more rational therapeutic approaches [28]. In particular, whole blood and serum-derived circulating predictive biomarkers of ICI response are of great interest because they are minimally invasive and may reflect overall tumor, as well as immune system-related changes occurring in response to ICIs [12,29]. In this regard, to explore phenotypic and molecular features of EVs in ICI responders and to identify novel candidate biomarkers of response, we used a novel approach, based on optimized PFC and shotgun proteomics, to characterize blood-derived EV subpopulations and protein cargo in patients with stage IV NSCLC [5,17,18,19,20,25].

In our study, low levels of total pre-treatment circulating EV were correlated with response to chemotherapy, but these levels were not correlated with overall survival in the corresponding cohort of patients. Conversely, low levels of EVs with endothelial phenotype (CD41a−/CD31+/CD45−) were consistently correlated with response to immunotherapy and with longer overall survival in patients treated with anti-PD-1/PD-L1 agents. Analysis of circulating EVs with leukocyte phenotype did not reveal any prognostic and predictive value for this EV subset in both patient cohorts. Intriguingly, CD31, one of the markers used to identify endothelial-derived vesicles, also known as platelet endothelial adhesion molecule-1 (PECAM-1), was shown to act as nonredundant co-modulator of T-cell responses and co-inhibitory receptor of dendritic cells [30]. In particular, CD31-deficient mice show enhanced T-cell mediated tumor rejection, associated to increased T cell-mediated killing and to decreased suppressive activity of Tregs [30,31]. Thus, the low levels of endothelial-derived CD31 positive EVs that we observed in ICI responders are in line with activation of immune response in these patients, whereas high levels of these EVs might reflect a mechanism of endothelial-mediated tumor immune escape occurring in non-responders [30,32]. Moreover, increased release of endothelial-derived EVs is known to occur due to endothelial dysfunction and enhanced angiogenesis that was shown to be frequently paralleled by immunosuppression in tumors [32,33,34,35,36,37]. Therefore, higher level of blood circulating endothelial-derived EVs may reflect the co-existence of a pro-angiogenic and immunosuppressive TME in tumors of non-responders to immunotherapy. Considering that neo-angiogenesis and immunosuppression are involved in modulating response to ICIs and that players in these pathways (e.g., IL6, vascular endothelial growth factor A [VEGFA], indoleamine 2,3-dioxygenase [IDO]) may be pharmacologically modulated, it will be interesting to study whether drugs acting on these pathways may improve ICI response [38]. Overall, the EV-phenotype that we observed in our single-center observational prospective characterization of blood circulating EVs shows that endothelial-derived EVs were related to ICI response and overall survival. Bootstrap internal validation procedure supported the reliability of our observations, however, further validation of these findings in a wider possibly multicentric population of NSCLC will be necessary.

Since the proteomic profile of EV cargos in ICI responders and non-responders with advanced NSCLC at baseline and on-treatment is not known, we performed an exploratory proteomic analysis of EV content in these patients. Proteomics showed different baseline and on-treatment modification of EV protein cargo between NSCLC responders and non-responders to anti-PD-1 therapy. Comparison of EV protein cargos at baseline between patients who responded to immunotherapy and patients who progressed during treatment, revealed a group of 10 proteins that were present only in EVs of responders. Some of these proteins, such as Annexin A2 and S100A9, are involved in regulation of immune escape in some cancer types, including lung cancer [39,40,41,42]. Unfortunately, transmembrane EV proteins such as CD31 were not detectable due to their relatively low abundance and hydrophobic properties that hamper their identification. Of note, in this study, Annexin A2 and S100A9 resulted differently modulated during immunotherapy between responders and non-responders and may represent candidate EV-related biomarkers. It is important to point out that fluorescence activated cell sorter isolates highly purified material, but this technique does not yield large numbers of EVs, suitable for some types of analyses (e.g., western blotting). This represents a technical limitation of the PFC protocol used to explore the proteome in responders and non-responders. Future studies with larger series and complementary techniques will be necessary to verify the predictive value of candidate EV-related biomarkers identified by our exploratory proteomic analysis.

## 5. Conclusions

In this report, immunophenotypical characterization of circulating EVs indicated that a low blood concentration of endothelial-derived EVs was associated with response to immune checkpoint blockers. Additionally, this study provided evidence that patients responding to immunotherapy had a specific EV protein cargo at baseline by proteomic analysis and that this cargo was deregulated after immunotherapy. These proteomic changes were previously unknown and warrant further investigations to verify which are the proteins more consistently deregulated in EV cargos of patients who benefit from anti PD-1 therapy. Overall, these results provide insights into immunophenotype and composition of EVs, which may pave the way to the identification of novel circulating biomarkers for improved immunotherapy.

## Figures and Tables

**Table 1:** Univariate and multivariate Cox proportional hazards regression analysis of survival in the immunotherapy cohort (*n* = 32).

		Univariate		Bootstrap Results (1000 Replicas)				Multivariate ^1^		Bootstrap Results (1000 Replicas)			
Variable	Groups.	HR (95% CI)	*p*	Bias	SE	95% CI	*p*	HR (95% CI)	*p*	Bias	SE	95% CI	*p*
Total EVs	≤14,360 EVs/μL vs. >14,360 EVs/μL^2^	0.45 (0.17–1.14)	0.09	−0.003	0.43	−1.70 to 0.01	0.04						
Leukocyte-EVs	≤169 EVs/μL vs.>169 EVs/μL ^2^	1.19 (0.26–2.01)	0.76	−0.01 ^3^	0.72 ^3^	−1.18 to 1.37 ^3^	0.72 ^3^						
Endothelial-EVs	≤94 EVs/μL vs. >94 EVs/μL ^2^	0.13 (0.04–0.50)	0.003	−0.19	0.91	−4.77 to −0.87	0.004	0.16 (0.04–0.63)	0.008	−0.96	3.10	−13.4 to −0.65	0.005
Age	≥65 vs. <65	1.24 (0.49–3.10)	0.65	0.06	0.47	−0.64 to 1.26	0.61						
No. metastatic sites	≥2 vs. <2	2.86 (1.02–8.04)	0.04	0.11	0.57	0.21 to 2.42	0.02	2.67 (0.73–9.70)	0.13	0.58	2.30	0.08 to 12.3	0.04
ECOG PS	1–2 vs. 0	2.77 (0.90–8.54)	0.08	0.14	0.64	0.19 to 2.66	0.02						
Tissue PD-L1	≥1% vs. <1%	0.77 (0.42–1.45)	0.43	−0.13	0.48	−1.67 to 0.30	0.45						
Line of therapy	2nd/3rd line vs. 1st line	1.18 (0.55–2.56)	0.66	−0.007	0.40	−0.70 to 0.97	0.40						

^1^ Variables with *p* < 0.05 in the univariate analysis were included in the multivariate analysis. ^2^ Optimal cut-off values derived from ROC curves. ^3^ Based on 999 samples Abbreviations: HR: Hazard ratio; SE: standard error; CI: confidence interval.

## Data Availability

The data presented in this study are available on request from the corresponding authors.

## Supplementary Materials

The following are available online at https://www.mdpi.com/2072-6694/13/4/585/s1, Figure S1: Workflow scheme of the experimental FACS-sorting-proteomics strategy used for the study; Figure S2: Kaplan-Meier (KM) curves showing the relationship between overall survival and pre-treatment blood levels of circulating total and leukocyte-derived EVs in the overall NSCLC population (a,d), immunotherapy cohort (b,e), and chemotherapy cohort (c,f); Figure S3: Changes in total, leukocyte- and endothelial-derived EV concentration during treatment (a,b,c) and according to immunotherapy response (d,e,f); Figure S4: Venn diagram of proteins quantified in pooled EVs extracted from cancer patients at their baseline divided in: Responders (RB) (blue) and Non-Responders (NRB) (pink); Figure S5: Relative expression (fold change in LOG scale) analysis of 10 EV-proteins obtained by comparing baseline and follow-up proteomics data in Responders (R) and Non-Responders (NR) patients; Figure S6: Functional Comparison Analysis of activated and inhibited pathways in responder (R) and non-responders’ (NR) sorted EVs compared to their respective baselines; Figure S7: Functional Comparison Analysis of activated and inhibited pathways in responder (R) and non-responders’ (NR) sorted EVs compared to their respective baselines; Figure S8: IPA networks legend. The figure shows the colour and shape key for IPA networks. Table S1: List of flow cytometry specificities and reagents; Table S2: NSCLC patients’ characteristics; Table S3: Analysis of pre-treatment EV concentration; Table S4: Comparison of total and endothelial-derived EV concentration between overall NSCLC population (n = 59) and healthy controls (n = 27) Table S5: Analysis of blood EV concentration at baseline and at follow-up (12 +/− 6 weeks) after day 1 of therapy in NSCLC patients treated with ICIs; Table S6: Univariate and multivariate Cox proportional hazards regression analysis of survival in the overall population (n = 59) and in the chemotherapy cohort (n = 28). Table S7: Distribution of clinical variables between groups with high and low endothelial EV blood concentration in the immunotherapy cohort (n = 26), Table S8: Comparison of median blood circulating EV concentration at baseline according to treatment response, Table S9: Protein identification: List of the identified proteins in the sorted-EVs from whole blood of healthy controls (HC) and lung cancer patients divided in responders (R) and non-responders (NR), respectively sorted at baseline (RB and NRB) and post-treatment (RP and NRP), Table S10: Identified protein in extracellular vesicles before treatment. Table shows the proteins identified in NRB EVs and RB EVs, Table S11: Functional analysis: List of the main downstream and upstream regulators obtained through Ingenuity Pathways Analysis (IPA) after Comparison of the single Core Analyses, Table S12: List of enriched Gene Ontology (GO) pathways and functionally related protein groups obtained by STRING analysis.

## Abbreviations

APC	Allophycocyanin
AUC	Curve with corresponding area under the curve
CR	Complete response
DCR	Disease control rate
EV	Extracellular vesicle
FASP	Filter-aided sample preparation
FMO	Fluorescence minus one
iBAQ	Intensity-based absolute quantification
ICI	Immune checkpoint inhibitor
IDO	Indoleamine 2,3-dioxygenase
IPA	Ingenuity Pathway Analysis
LC-MS/MS	Liquid chromatography tandem mass spectrometry
LCD	Lipophilic cationic dye
MBR	Match-between-runs
mOS	Median overall survival
NRB	Non-Responders at baseline
NRP	Non-responders post-treatment
NSCLC	Non-small cell lung cancer
PD-1	Programmed cell death 1
PD-L1	Programmed cell death-ligand 1
PD	Progressive disease
PECAM-1	Platelet endothelial adhesion molecule-1
PFC	Polychromatic flow cytometry
PR	Partial response
RB	Responders at baseline
ROC	Receiving operator characteristic
RP	Responders post-treatment;
SD	Stable disease;
TME	Tumor microenvironment;
URA	Upstream Regulator Analysis;
VEGF	Vascular endothelial growth factor;
