# Supplementary material for: Phenotypic and Proteomic Analysis Identifies Hallmarks of Blood Circulating Extracellular Vesicles in NSCLC Responders to Immune Checkpoint Inhibitors
Source: Cancers (Basel). 2021 Feb 3;13(4):585. doi: 10.3390/cancers13040585 (PMC7913165; doi:10.3390/cancers13040585)
Supplement: Supplementary file 1 [file cancers-13-00585-s001.zip › cancers-1075777-SUPPL-FINAL.pdf]

Supplementary Materials: Phenotypic and Proteomic Analysis Identifies Hallmarks of Blood Circulating Extracellular Vesicles in NSCLC Responders to Immune Checkpoint Inhibitors

Davide Brocco, Paola Lanuti, Damiana Pieragostino, Maria Concetta Cufaro, Pasquale Simeone, Giuseppina Bologna, Pietro Di Marino, Michele De Tursi, Antonino Grassadonia, Luciana Irtelli, Laura De Lellis, Serena Veschi, Rosalba Florio, Luca Federici, Marco Marchisio, Sebastiano Miscia, Alessandro Cama, Nicola Tinari and Piero Del Boccio

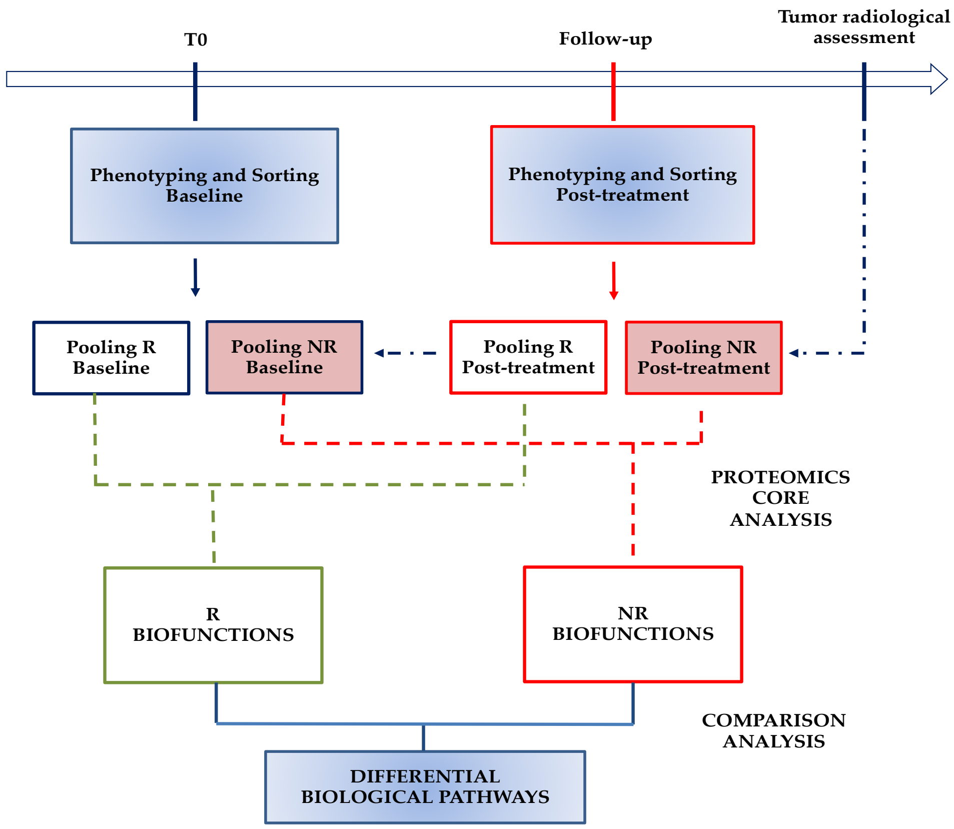

Figure S1. Workflow scheme of the experimental FACS-sorting-proteomics strategy used for the study. EVs sorted at baseline and during anti-PD-1 immunotherapy were analyzed through shotgun mass spectrometry analysis for simultaneous identification and quantification of proteins.

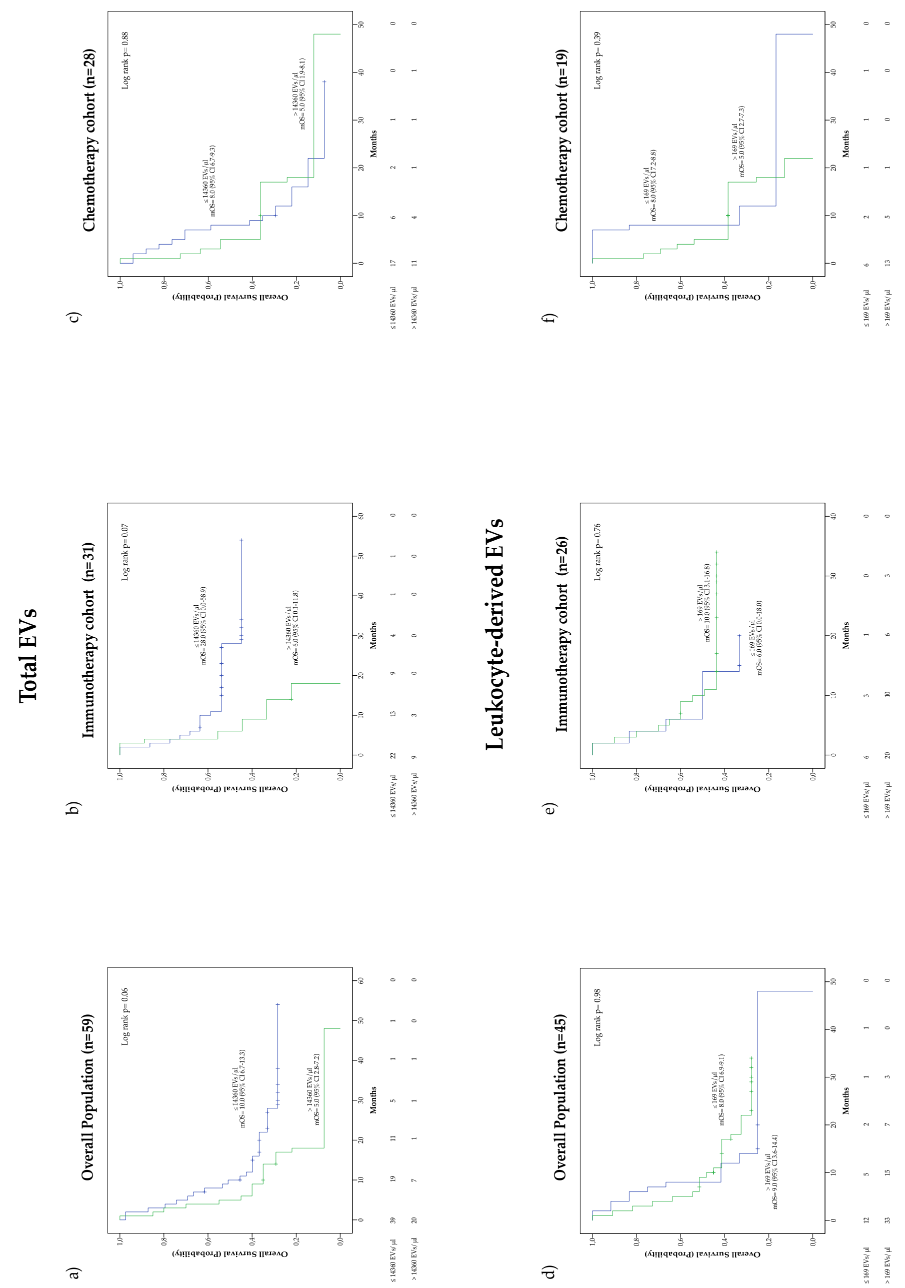

Figure S2. Kaplan-Meier (KM) curves showing the relationship between overall survival and pre-treatment blood levels of circulating total and leukocyte-derived EVs in the overall NSCLC population **(a,d)**, immunotherapy cohort **(b,e),** and chemotherapy cohort **(c,f)**.

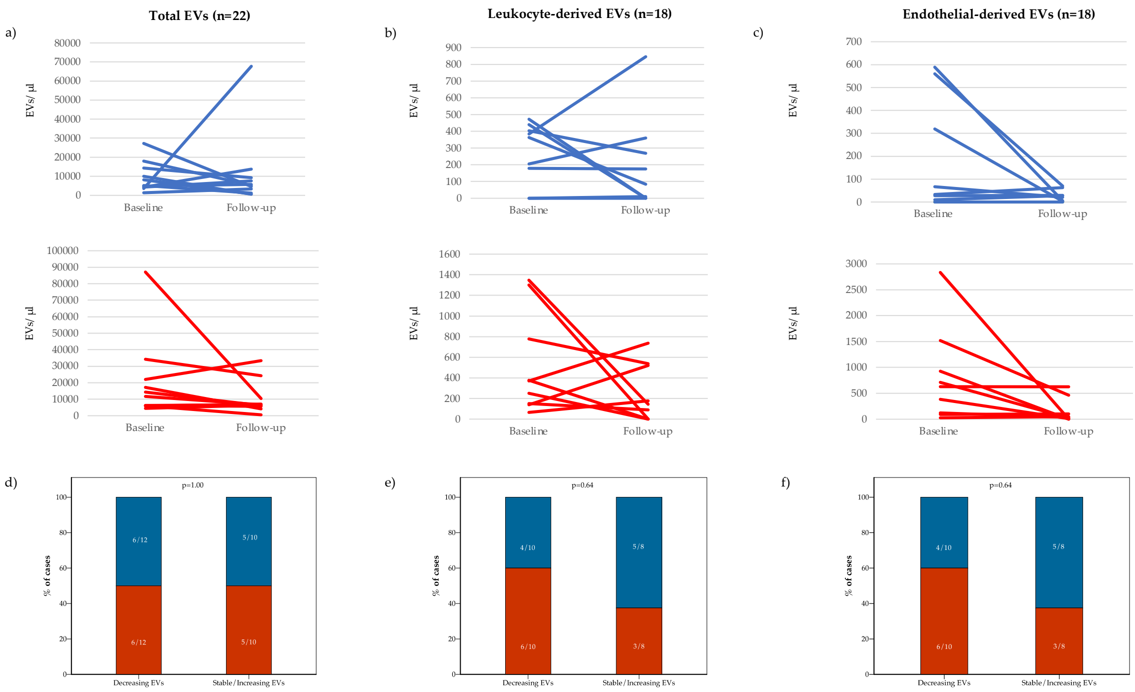

Figure S3. Changes in total, leukocyte− and endothelial−derived EV concentration during treatment **(a,b,c)** and according to immunotherapy response **(d,e,f).** Changes in EV concentration between baseline and follow−up are indicated in blue for responders and in red for non−responders. Fisher's exact test was used to compare the association between changes in EV concentration and treatment response.

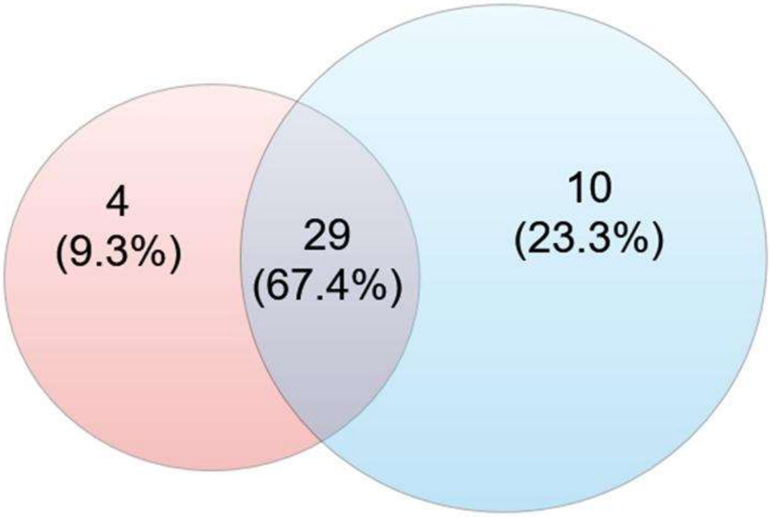

Protein	NRB	RB
AZGP1	+	
CA2	+	
CA1	+	
HBD	+	
CAT		+
S100A9		+
DSC1		+
ANXA2		+
JUP		+
LYZ		+
IGHG2		+
ORM2		+
HPX		+
IGHV3OR16-9		+

Figure S4. Venn diagram of proteins quantified in pooled EVs extracted from cancer patients at their baseline divided in: Responders (RB) (blue) and Non−Responders (NRB) (pink). Table shows the unique proteins of the two groups (R and NR) at the baseline. Ten proteins were detected only in pre−treatment EVs of responding patients. In particular the RB specific proteins were: Catalase (CAT), S100 calcium−binding protein A9 (S100−A9), Desmocollin−1 (DSC1), Annexin A2 (ANXA2), Lysozyme C (LYZ), Junction plakoglobin (JUP), Immunoglobulin heavy constant gamma 2 (IGHG2), Alpha−1−acid glycoprotein 2 (ORM2), Immunoglobulin heavy variable 3/OR16−9 (IGHV3OR16−9)

and Hemopexin (HPX). The four proteins specifically identified in NRB were: Zinc−alpha−2−glycoprotein (AZGP1), Carbonic anhydrase 1 and 2 (CA1) and (CA2), Hemoglobin subunit delta (HBD).

a)

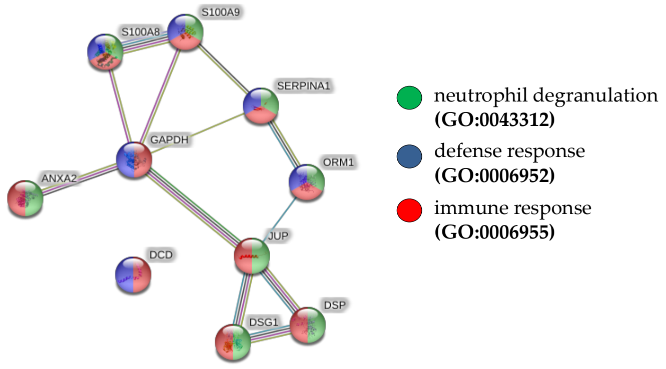

b)

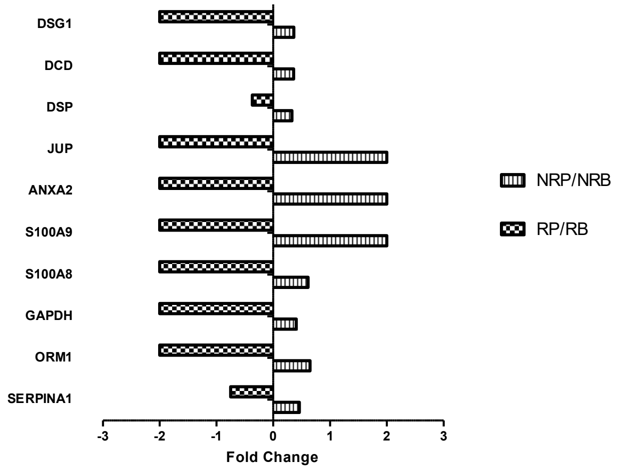

Figure S5. Relative expression (fold change in LOG scale) analysis of 10 EV−proteins obtained by comparing baseline and follow−up proteomics data in Responders (R) and Non−Responders (NR) patients. (**a**) Network of interactions obtained by STRING analysis (https://string−db.org/) of these proteins. Gene Ontology Classification of the proteins and their color functional enrichment were reported in the legend. Panel (**b**) shows a trend for a decreased expression of all EV proteins during immunotherapy in responders. In contrast, a positive fold−change was observed in patients with cancer, which failed to achieve a response.

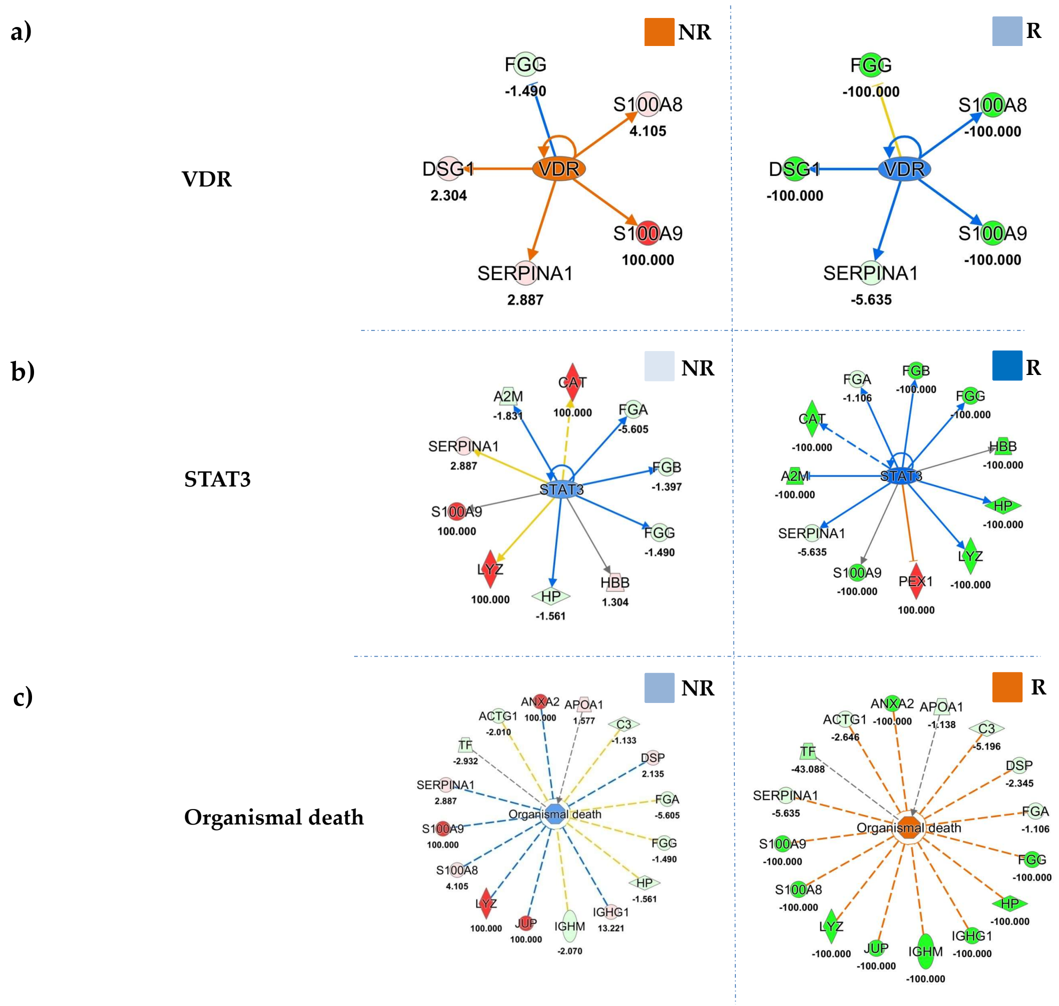

Figure S6. Functional Comparison Analysis of activated and inhibited pathways in responder (R) and non−responders' (NR) sorted EVs compared to their respective baselines. Panel A reports the upstream analysis in the two proteomics conditions analyzed for Vitamin D Receptor (VDR). Panel B shows STAT3 modulation in NR and R EVs. The Panel C reports mild activation of the "Organismal death" (z−score = 3.58) only in EVs of responding patients. The intensity of each activated (orange) and Inhibited (blue) z−scores are reported in the squares of the heatmaps for each comparison. Red and green shapes represent increased or decreased measurements of identified proteins, respectively, whose fold change value is reported in figure. Colour key and symbols are reported in Figure S8.

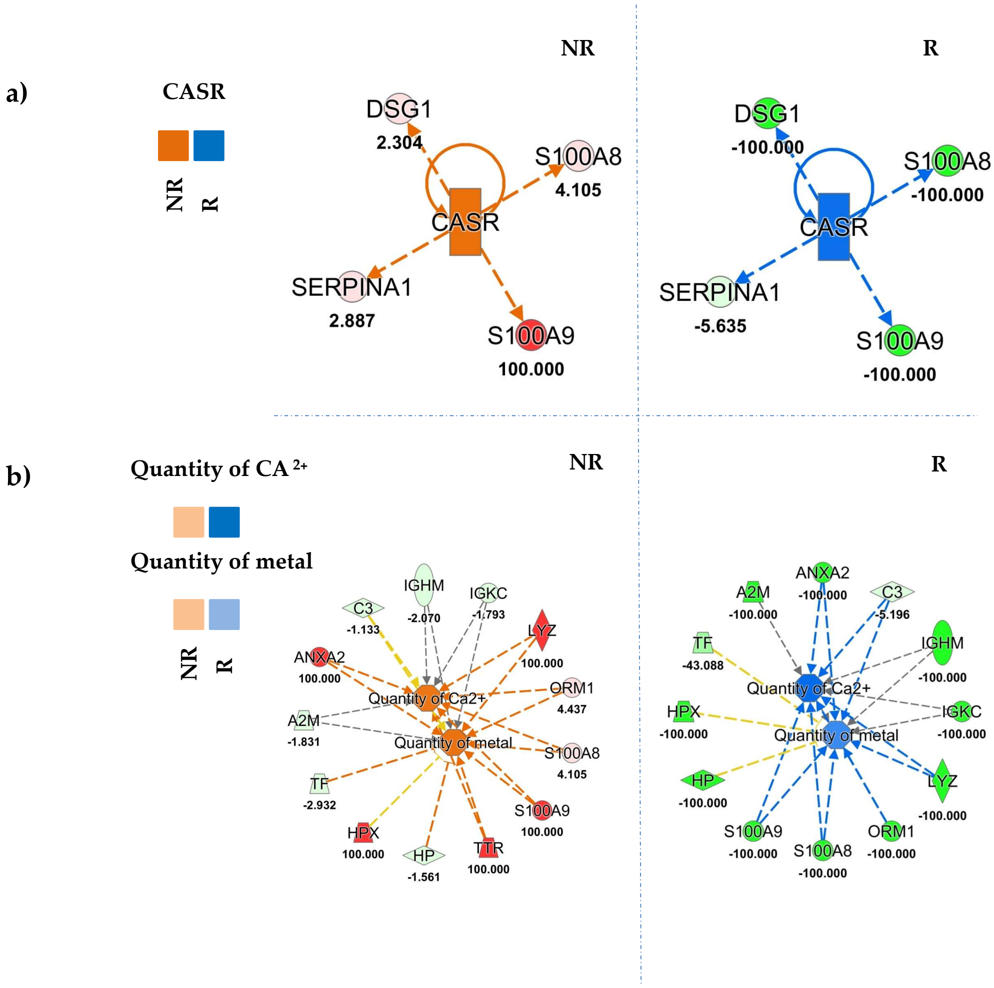

Figure S7. Functional Comparison Analysis of activated and inhibited pathways in responder (R) and non−responders' (NR) sorted EVs compared to their respective baselines. In Panel A, EVs protein cargo analysis revealed activation (z−score = 2.72) of Calcium Sensing Receptor (CASR) in NRP and its inhibition (z−score = −2.0) in responding patients. In Panel B, the same comparison is reported for two functions: "Quantity of CA^{2+}" and "Quantity of metals". The modulation of CASR was in line with the downstream inhibition of "Quantity of metals" and "Quantity ofCa^{2+}". The intensity of each activated (orange) and Inhibited (blue) z−scores are reported in the squares of the heatmaps for each comparison. Red and green shapes represent increased or decreased measurements of identified proteins, respectively, whose fold change value is reported in figure. Colour key and symbols are reported in Figure S8.

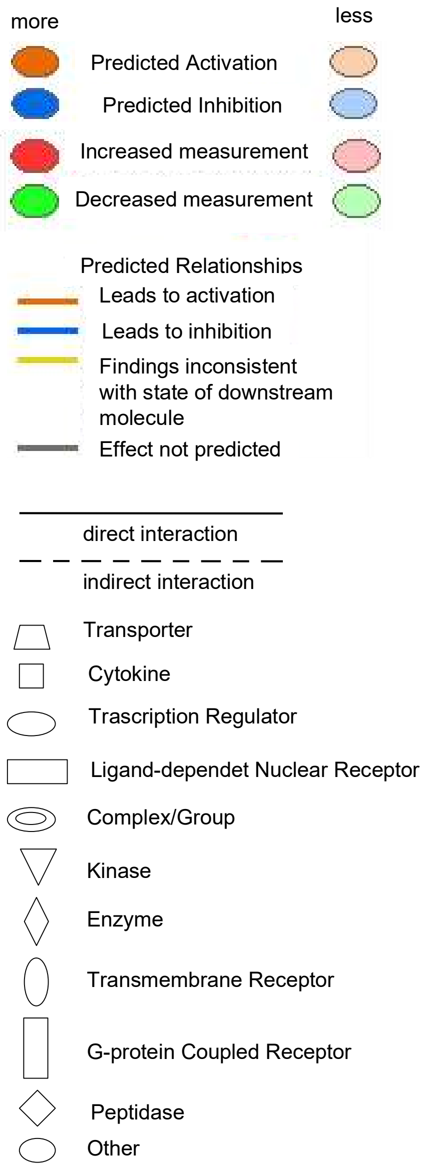

Figure S8. IPA networks legend. The figure shows the colour and shape key for IPA networks.

Table S1. List of flow cytometry specificities and reagents.

Reagent*	Fluorochrome/Reagent	Vendor	Clone	Cat. Number	Volume Per Test (µl)
Lipophilic Cationic Dye (LCD)	-	BD Biosciences	-	626267	0.5
Phalloidin-FITC	FITC	BD Biosciences	-	626267	0.5
CD41a	PE	BD Biosciences	HIP8	626266	5
CD31	PE-Cy7	BD Biosciences	WM59	626266	5
CD45	BV510	BD Biosciences	HI30	626266	5

FITC=Fluorescein isothiocyanate; PE= R-phycoerythrin; PE-Cy7= PE-Cyanine 7, BV=Brilliant Violet. * The reagent mix was prepared by adding the reagents to 195 µl of PBS 1X and 5 µl of whole blood. After 45 min of staining (RT, in the dark), 500 µl PBS 1X was added to each tube, and 1 × 106 events/ sample were acquired by flow cytometry (FACSVerse, BD Biosciences).

Table S2. NSCLC patients' characteristics.

Variable	ICIs (*n* = 31)	Chemotherapy (*n* = 28)	Overall (*n* = 59)
Age (%)			
≥65	18 (58.1)	22 (78.6)	40 (67.8)
<65	13 (41.9)	6 (21.4)	19 (32.2)
Sex (%)			
Male	24 (77.4)	23 (82.1)	47 (79.7)
Female	7 (22.6)	5 (17.9)	12 (20.3)
Tissue PD-L1 Expression (%)			
≥1%	23 (74.2)	6 (21.4)	29 (49.2)
<1%	6 (19.4)	12 (42.9)	18 (30.5)
Not evaluable	2 (6.5)	10 (35.7)	12 (20.3)
Number of metastatic sites (%)			
≥2	12 (38.7)	16 (57.1)	28 (47.5)
<2	19 (61.3)	12 (42.9)	31 (52.5)
Smoking (%)			
Current smoker	22 (71.0)	13 (46.4)	35 (59.3)
Never/Former smoker	7 (22.6)	12 (42.9)	19 (32.2)
Unknown	2 (6.5)	3 (10.7)	5 (8.5)
ECOG PS			
0	9 (29.0)	12 (42.9)	21 (35.6)
1-2	22 (71.0)	16 (57.1)	38 (64.4)
Prior systemic therapy for advanced disease			
None	18 (58.1)	25 (89.3)	43 (72.9)
ICIs	0	0 (0.0)	0 (0.0)
Chemotherapy	12 (38.7)	3 (10.7)	15 (25.4)
Other	1 (3.2)	0 (0.0)	1 (1.7)
Histology (%)			
Adenocarcinoma	24 (77.4)	19 (67.9)	43 (72.9)
Squamous cell carcinoma	7 (22.6)	9 (32.1)	16 (27.1)

Table S3. Analysis of pre-treatment EV concentration.

	Overall Population		Chemotherapy		Immunotherapy		
	n	**Median EVs/µl (CI 95%)**	*n*	**Median EVs/µl (CI 95%)**	*n*	**Median EVs/µl (CI 95%)**	***p*-Value***
**Total EV**	59	8414 (6647–14350)	28	8004 (6649–18847)	31	9435 (6045–14350)	0.35
**Leukocyte EVs**	45	362 (250–455)	19	304 (169–748)	26	380 (251–511)	0.53
**Endothelial EVs**	45	146 (73–385)	19	235 (94–750)	26	116 (39–385)	0.31

* Mann-Whitney test was performed to compare median EVs/µl between chemotherapy and immunotherapy.

Table S4. Comparison of total and endothelial-derived EV concentration between overall NSCLC population (*n* = 59) and healthy controls (*n* = 27).

	**NSCLC**	**HCs**	**p-value**
Age (%)			
≥65	40 (67.8)	14 (51.9)	0.23
<65	19 (32.2)	13 (48.1)	
Sex (%)			
Male	47 (79.7)	16 (59.3)	0.06
Female	12 (20.3)	11 (40.7)	
Median Total EVs/µl (95% CI) (*n* = 59)	8414 (6647-14350)	4045 (2503-6243)	0.00001
Median Endothelial-derived EVs/µl (95% CI) (*n* = 45)	146 (73-385)	62 (42-107)	0.03

Table 5. Analysis of blood EV concentration at baseline and at follow-up (12 +/- 6 weeks) after day 1 of therapy in NSCLC patients treated with ICIs.

	**Baseline**	**Follow-Up**	***p*-Value***

	n	**Median EVs/µL (CI 95%)**	*n*	**Median EVs/µl (CI 95%)**	
Total EVs/µL	31	9435 (6045–14350)	22	6305 (4670–7863)	0.051
Leukocyte EVs/µL	26	380 (251–511)	22	117 (1–242)	0.008
Endothelial EVs/µL	26	116 (39–385)	22	29 (5–67)	0.017

* Mann Whitney test was performed to compare median EVs/µl between baseline and follow-up EV concentrations.

Table S6. Univariate and multivariate Cox proportional hazards regression analysis of survival in the overall population (*n* = 59) and in the chemotherapy cohort (*n* = 28).

–		**Overall Population**											
		**Univariate**		**Bootstrap Results (1000 Replicas)**				**Multivariate***		**Bootstrap Results (1000 Replicas)**			
**Variable**	**Groups**	**HR (95% CI)**	***p***	**Bias**	**SE**	**95 % CI**	***p***	**HR (95% CI)**	***p***	**Bias**	**SE**	**95 % CI**	***p***
Total EVs.	≤14,360 EVs./µL vs. >14,360 EVs./µL	0.57 (0.31-1.04)	0.07	0.02	0.30	−1.14 to 0.4	0.050						
Leukocyte−EVs.	≤169 EVs./µL vs. >169 EVs./µL	1.01 (0.46−2.20)	0.98	0.009	0.38	−0.74 to 0.77	0.98						
Endothelial−EVs.	≤94 EVs./µL vs. >94 EVs./µL	**0.39 (0.17−0.86)**	**0.02**	−0.029	0.40	−1.77 to −0.24	**0.02**	**0.40 (0.19−0.89)**	**0.03**	−0.03	0.46	−1.92 to −0.12	**0.03**
Age	≥65 vs. <65	1.32 (0.70−2.50)	0.39	0.05	0.31	−0.23 to 1.01	0.33						
No. metastatic sites	≥2 vs. <2	1.59 (0.87−2.91)	0.13	0.01	0.31	−0.12 to 1.10	0.13						
ECOG PS	1−2 vs. 0	**2.42 (1.23−4.78)**	**0.01**	0.04	0.34	0.29 to 1.63	**0.006**	**2.38 (0.16−0.96)**	0.059	0.08	0.62	0.06 to 2.01	**0.04**
Tissue PD−L1	≥1% vs. <1%	0.75 (0.52−1.10)	0.14	−0.01	0.22	−0.76 to 0.13	0.18						
Line of therapy	2nd/3rd line vs. 1st line	0.80 (0.43−1.50)	0.50	0.005	0.30	−0.85 to 0.34	0.47						
		**Chemotherapy**											

		Univariate		Bootstrap Results (1000 Replicas)				Multivariate		Bootstrap Results (1000 Replicas)			
**Variable**	**Groups**	**HR (95% CI)**	***p.***	**Bias**	**SE**	**95 % CI**	***p.***	**HR (95% CI)**	***p.***	**Bias**	**SE**	**95 % CI**	***p.***
Total EVs.	≤14350 EVs./μL vs. >14350 EVs./μL	0.94 (0.41–2.17)	0.88	−0.03 [a]	0.53 [a]	−1.18 to 0.92 [a]	0.90 [a]						
Leukocyte−EVs.	≤169 EVs./μL vs. >169 EVs./μL	0.64 (0.22–1.87)	0.41	−0.04[b]	0.71[b]	−2.72 to 0.57[b]	0.36[b]						
Endothelial−EVs.	≤94 EVs./μL vs. >94 EVs./μL	1.67 (0.58–4.89)	0.34	0.04[c]	0.62[c]	−0.37 to 1.69[c]	0.22[c]						
Age	≥65 vs. <65	0.67 (0.46–3.33)	0.67	−0.07	0.59	−0.86 to 1.35	0.66						
No. metastatic sites	≥2 vs. <2	1.14 (0.50–2.60)	0.75	0.08	0.48	−0.72 to 1.22	0.74						
ECOG PS	1−2 vs. 0	2.54 (1.07–6.04)	**0.04**	0.07	0.55	0.07 to 2.26	**0.03**						
Tissue PD−L1	≥1% vs. <1%	1.03 (0.56–1.91)	0.91	−0.06	0.38	−0.82 to 0.73	0.38						
Line of therapy	2nd/3rd line vs. 1st line	0.76 (0.18–3.29)	0.71	−0.23[d]	1.06[d]	−3.20 to 0.78[d]	0.69[d]						

* Variables with $P < 0.05$ in the univariate analysis were included in the multivariate analysis. (**a**) Based on 998 samples; (**b**) Based on 999 samples; (**c**) Based on 997 samples; (**d**) Based on 951 samples;.

Table S7. Distribution of clinical variables between groups with high and low endothelial EV blood concentration in the immunotherapy cohort (*n* = 26).

	**High Endothelial EV Concentration**		**Low Endothelial EV Concentration**		
**Variable**	**Number (*n* = 14)**	**Percentage %**	**Number (*n* = 12)**	**Percentage %**	***p*–Value**
Age (years)					0.42
≥65	10	71.4	6	50.0	
<65	4	28.6	6	50.0	
Sex					1.00
Male	11	78.6	10	83.3	
Female	3	21.4	2	16.7	
Line of therapy					0.52
1	7	50.0	8	66.7	
2	6	42.9	4	33.3	
≥3	1	7.1	0	0.0	
ICIs					**0.06**
Nivolumab	3	21.4	0	0.0	
Pembrolizumab	7	50.0	11	91.7	
Atezolizumab	4	28.6	1	8.3	
Number of metastatic sites					0.42
≥2	10	71.4	6	50.0	
<2	4	28.6	6	50.0	
Histology (%)					0.27
Adenocarcinoma	9	64.3	10	83.3	
Squamous cell carcinoma	5	35.7	2	16.7	
ECOG PS					0.37
0	2	14.3	4	33.3	
1–2	12	85.7	8	66.7	
Tissue PD–L1 Expression (%)					
≥1%	8	57.1	11	91.7	0.13
<1%	5	28.6	1	16.7	
Not evaluable	1	14.3	0	0.0	

Table S8. Comparison of median blood circulating EV concentration at baseline according to treatment response.

		Overall Population			Chemotherapy			Immunotherapy		
		n	**Median EVs/µL (CI 95%)**	***p*.***	***n***	**Median EVs/µL (CI 95%)**	**p.***	***n***	**Median EVs/µL (CI 95%)**	***p*.***
**Total EVs**	Responders	28	6994 (5616– 9723)	**0.03**	15	6944 (6037–13656)	**0.03**	13	8179 (4312–12667)	0.16
	Non–Responders	29	14017 (6936–21897)		12	19235 (8998–61834)		17	11695 (6045–20000)	
**Leukocyte EVs**	Responders	21	362 (179–579)	0.95	10	331 (161–840)	0.46	11	384 (179–472)	0.48
	Non–Responders	24	350 (250–584)		9	265 (190–544)		15	376 (151–837)	
**Endothelial EVs**	Responders	21	39 (20–319)	**0.02**	10	192 (17–751)	0.46	11	33 (10–319)	**0.01**
	Non–Responders	24	280 (109–628)		9	235 (99–4035)		15	324 (102–671)	

*Mann–Whitney test was performed to compare median EVs/µl between responders and non responders.

Table S9. Protein identification: List of the identified proteins in the sorted−EVs from whole blood of healthy controls (HC) and lung cancer patients divided in responders (R) and non responders (NR), respectively sorted at baseline (RB and NRB) and post−treatment (RP and NRP). Table reports the raw data, the protein quantification and the fold change for each defined comparison.

(see uploaded file: Supplementary Table 9_Protein identification.xlsx).

Table S10. Identified protein in extracellular vesicles before treatment. Table shows the proteins identified in NRB EVs and RB EVs. The sign "+" indicates the presence into the specific EV group.

Protein	NRB	RB
A2NJV5	+	+
P04406	+	+
Q02413	+	+
P05109	+	+
P15924	+	+
P81605	+	+
Q5T749	+	+
P00738	+	+
P02763	+	+
P63261	+	+
P02787	+	+
P01876	+	+
P68104	+	+
P01857	+	+
P01834	+	+
P01009	+	+
P01860	+	+
P01023	+	+
P0DP03	+	+
P01871	+	+
P02647	+	+
P01024	+	+
P02675	+	+
P02679	+	+
P02671	+	+
P68871	+	+
P69905	+	+
P25311	+	−
P00918	+	−
P00915	+	−
P02042	+	−
P04040	−	+
P06702	−	+
Q08554	−	+
P07355	−	+
P35326	−	+
P14923	−	+
P61626	−	+
P01859	−	+
P19652	−	+
P0DOY3	−	+
P02790	−	+
A0A0B4J2B5	−	+

Table S11. Functional analysis: List of the main downstream and upstream regulators obtained through Ingenuity Pathways Analysis (IPA) after Comparison of the single Core Analyses. Table reports the upstream regulator or the disease and function annotation, its predicted activation state, its p-value of overlap, its z-score activation and the target molecules of the dataset. (see uploaded file: Supplementary Table 11_Functional analysis.xlsx)

Table S12. List of enriched Gene Ontology (GO) pathways and functionally related protein groups obtained by STRING analysis. (see uploaded file: Supplemental Table 12_List of Enriched Gene Ontology (GO) Pathways.xlsx)
